# Theoretical Challenges in Polaritonic Chemistry

**DOI:** 10.1021/acsphotonics.1c01749

**Published:** 2022-02-15

**Authors:** Jacopo Fregoni, Francisco J. Garcia-Vidal, Johannes Feist

**Affiliations:** Departamento de Física Teórica de la Materia Condensada and Condensed Matter Physics Center (IFIMAC), Universidad Autónoma de Madrid, 28049 Madrid, Spain

**Keywords:** molecular polaritons, strong coupling, photochemistry, nanoplasmonics, resonant cavities, cavity-QED

## Abstract

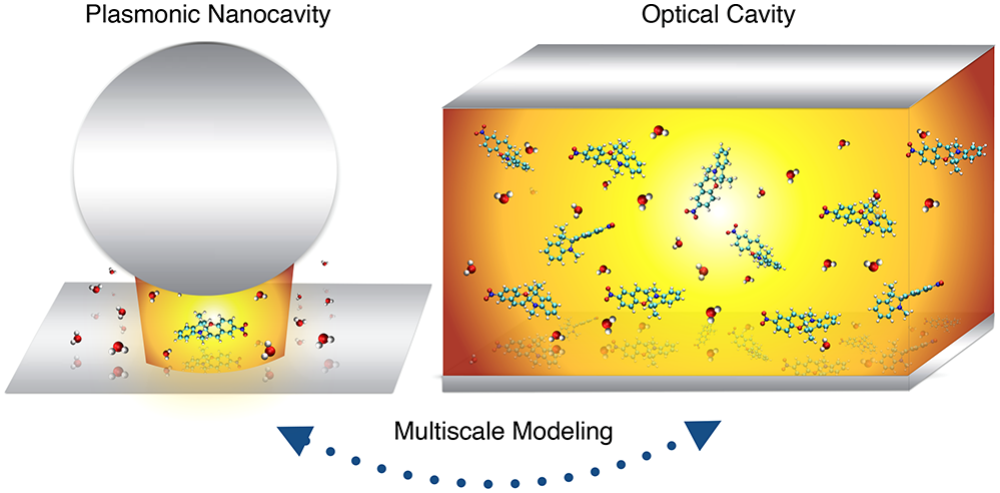

Polaritonic chemistry exploits strong light–matter coupling between molecules and confined electromagnetic field modes to enable new chemical reactivities. In systems displaying this functionality, the choice of the cavity determines both the confinement of the electromagnetic field and the number of molecules that are involved in the process. While in wavelength-scale optical cavities the light–matter interaction is ruled by collective effects, plasmonic subwavelength nanocavities allow even single molecules to reach strong coupling. Due to these very distinct situations, a multiscale theoretical toolbox is then required to explore the rich phenomenology of polaritonic chemistry. Within this framework, each component of the system (molecules and electromagnetic modes) needs to be treated in sufficient detail to obtain reliable results. Starting from the very general aspects of light–molecule interactions in typical experimental setups, we underline the basic concepts that should be taken into account when operating in this new area of research. Building on these considerations, we then provide a map of the theoretical tools already available to tackle chemical applications of molecular polaritons at different scales. Throughout the discussion, we draw attention to both the successes and the challenges still ahead in the theoretical description of polaritonic chemistry.

Ever since the invention of the first lasers,^1^ the role of light in modern chemistry has been to act either as a probe or as a trigger to respectively explore and induce photophysical and photochemical events. Over the last years, a complementary paradigm based on the use of confined light modes in micro- and nanocavities has been developed. Here, the confinement enhances the interaction between the quantum states of light and the molecular transitions to such an extent that the so-called strong-coupling regime is entered, and the excited states of the system become hybrids between light and matter, known as polaritons. Polaritons inherit properties from both their constituents and also possess new properties due to their hybrid nature, potentially leading to significant changes in the photophysics and photochemistry of the coupled systems. The interest in strong coupling for modifying chemistry arose almost a decade ago after a seminal experiment showed that photochemical reaction rates can be modified in cavities.^2,3^

This new direction to modify and control the properties of molecular systems is nowadays known as polaritonic chemistry.^4−6^ It has been shown to affect a wide range of processes, such as photochemical reactions both in single-molecule^7−14^ and collective^2,3,15−20^ strong-coupling setups, as well as (possibly long-range) energy transfer^21−31^ and transitions between different spin multiplets,^32−39^ among others. We emphasize that polaritonic chemistry is not a mere substitute for traditional chemistry techniques, as it can enable processes that are not possible in bare materials due to the long-range and collective nature of the polaritons. We also stress that, while polariton formation can affect many different processes, this should not be misunderstood as a binary switch with no changes in the weak coupling regime and “full” changes in the strong coupling regime. On the one hand, the exact crossover point between these regimes is in any case somewhat arbitrary, especially in nonidealized systems and in the presence of disorder, and on the other hand, many effects depend on the interplay of many complex parts, with their magnitude depending on details of the energetic and state overlaps, vibrational mode structure, and so on. Consequently, a smooth behavior is to be expected as a function of the light–matter coupling strength, with some optimal value that is often clearly within the strong-coupling regime, but can also lie just at its onset.^11^

Despite the attractiveness of these applications and the large range of existing works, there are many open questions and fundamental problems that remain to be addressed. The goal of this Perspective is to provide an overview of and guide through the challenges facing theoretical treatments of polaritonic chemistry, which we hope will be useful as a guide both for scientists active in the field and those entering it. Fundamentally, these challenges are due to the large complexity of the studied systems, which manifests on multiple scales: the building blocks are (often organic) molecules, which locally interact with their environment and each other, as well as electromagnetic (EM) field modes that are usually highly lossy and possess complex mode structures. Both of these building blocks can be treated in arbitrary detail and possess a rich phenomenology. Consequently, the study of each type of subsystem in isolation is the topic of a large field of science (respectively, chemistry and (nano)photonics). Within polaritonic chemistry, these building blocks are made to interact strongly, and the resulting hybrid states, the polaritons, possess properties that are not found in either subsystem in isolation. Furthermore, in most experimentally relevant setups, there are important collective effects, with macroscopic numbers of molecules coupling to every single EM mode and, at the same time, many EM modes being involved. In order for collective coupling to arise, the molecules should have similar excitation energies (i.e., absorption spectra). This is trivially the case for identical molecules, but can also be achieved in other situations.^31,40^ Finally, the quantized nature of the EM fields often plays a major role, requiring the use of techniques from (cavity) quantum electrodynamics and quantum optics to achieve a faithful description of the systems. Due to the often highly lossy nature of the EM modes, these techniques typically have to be combined with those of open quantum systems.

The very general considerations above already imply that a full theoretical ab initio modeling of such systems is effectively impossible without significant approximations. The challenge thus lies in choosing the appropriate simplifications and approximations in each specific situation. At the same time, the huge available design space implies that the existing work up to now has only scratched the surface of what is possible, and there is considerable potential for future advancements. In order to maintain a manageable scope, in the current Perspective, we focus on “chemical” applications, that is, the treatment of (collections) of molecules in the presence of quantized EM modes, without discussing in detail how to obtain or design such modes, or uses of the coupled systems for photonics applications. Furthermore, we restrict ourselves for the most part to the situation where electronic transitions in the molecules are coupled to light modes. Recent years have also seen an explosion of activity in vibrational strong coupling, where (IR-active) transitions between vibrational states in the molecules are coupled to cavity modes. Several recent Perspectives and Reviews have treated such setups, and we encourage the interested reader to consult those.^6,41−46^

## Overview of Experimental Setups

**Figure 1:**
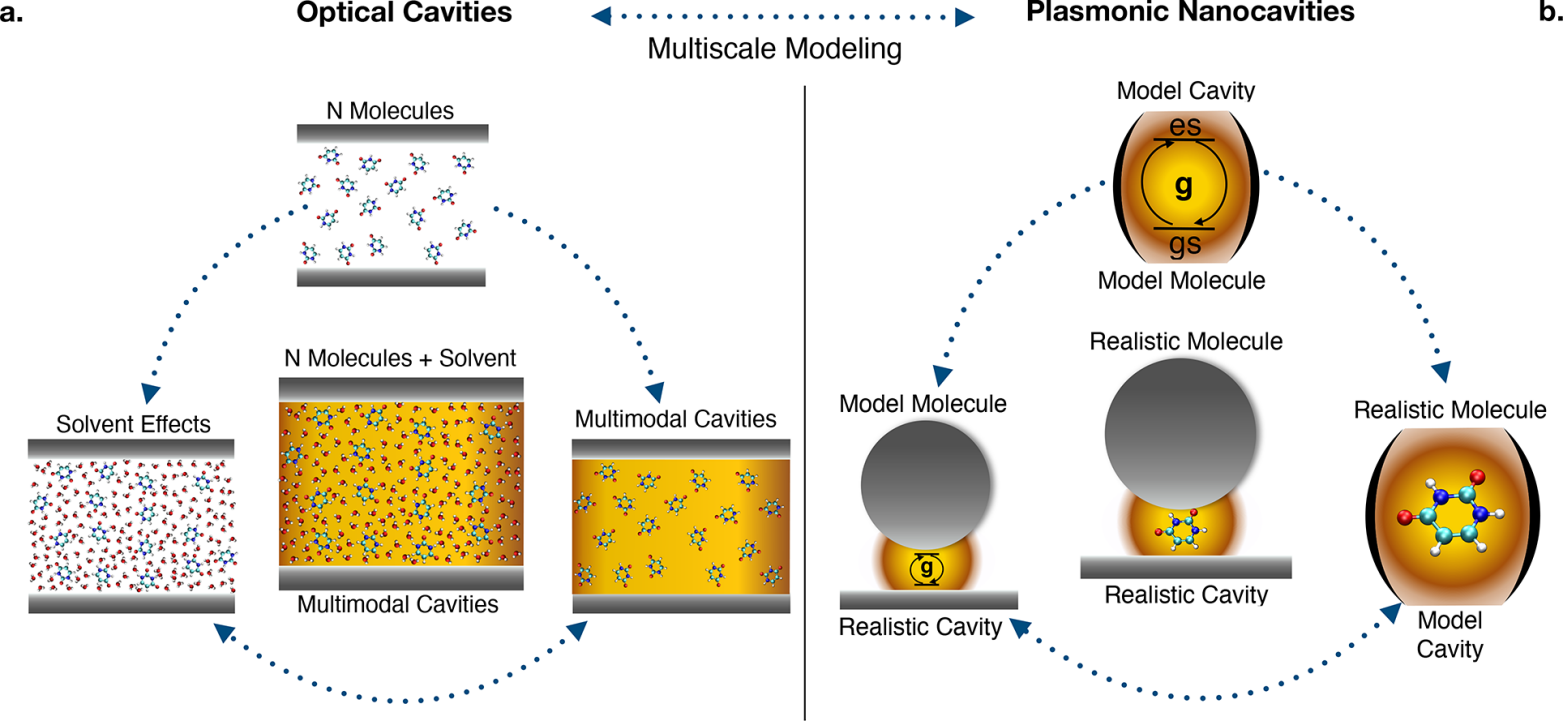
Polaritonic chemistry as a multiscale problem. (a) The challenges in modeling polaritonic chemistry in photonic cavities involve the description of large ensemble of molecules collectively coupled, all embedded in a complex chemical environment. (b) The challenges in achieving a detailed description in plasmonic nanocavities involve the accurate modeling of the plasmonic inhomogeneous electromagnetic field to be interfaced with an accurate quantum-chemical treatment of (relatively few) molecules.

In this section, we provide an overview of typical experimental setups that have been explored on the road toward polaritonic devices^47^ to control chemistry. Organic (often dye) molecules are commonly used, which have excitation energies of a few eV and line widths about an order of magnitude smaller (at room temperature). Most experiments can be categorized into one of two distinct groups that are distinguished by the photonic platform and the number of involved molecules (see Figure 1). The first are optical cavities, most often formed by planar mirrors (Fabry–Pérot cavities). The cavity modes are then standing waves with characteristic dimensions similar to the free-space wavelength. In such systems, strong coupling is achieved with macroscopic numbers of molecules,^2,48,49^ as depicted in Figure 1a. The relatively large size of such cavities means that fabrication is not too challenging and allows the use of liquid samples.^50^

The second type is subwavelength plasmonic (i.e., metallic) cavities, where the “light” modes are characterized by collective oscillations of the electrons in the structure, which permits the concentration of one quantum of excitation to spatial scales far below the free-space wavelength. While such systems are often referred to as (nano)cavities for simplicity, a more physically accurate nomenclature is “resonator” or “antenna”. Effective mode volumes (roughly proportional to the physical volume occupied by the EM mode) can reach below 100 nm^3^^51^ and possibly even down to ≈1 nm^3^.^52−55^ Such setups, depicted in Figure 1b, allow strong coupling to be reached with a few molecules^56,57^ or even a single emitter.^51,58−60^

Despite a difference of many orders of magnitude in the effective volume of the modes and the number of involved molecules, typical Rabi splittings (corresponding to the energy difference between the two polariton modes formed when a molecular transition and a cavity mode are on resonance) in both systems are comparable and range from Ω_R_ ≈ 100 meV up to more than an eV.^2,61,62^ At first sight, it might seem somewhat surprising that such physically different systems lead to similar effective coupling strengths, but this is actually straightforward to understand. To do so, we treat a simplified model of *N* identical two-level molecules (where only the lowest two electronic states are taken into account and rovibrational motion is ignored) that are all coupled identically to a single EM mode, such that the space-dependent electric field profile is ignored. In that situation, the Rabi splitting is given by^63^
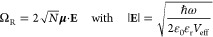
1where **μ** is the molecular transition dipole moment, *V*_eff_ is the effective mode volume of the confined EM field, ε_0_ is the vacuum permittivity, and ε_r_ is the relative background permittivity of the molecular material. The result is that , which implies that the Rabi splitting is proportional to the dipole density of the molecular material, but does not depend separately on the absolute number of molecules or volume of the cavity mode. In other words, large cavities give the same Rabi splitting as small ones because the per-molecule coupling decreases, but they can be filled with more molecules. A more detailed study shows that the Rabi splitting is proportional to the square of the dipole density times a scalar filling factor (ranging between 0 and 1) that measures the fraction of the photonic mode that is filled with the molecular material.^64,65^ The Rabi splitting can also be rewritten in terms of the amplitude of the molecular transition obtained when expressing the dielectric function of the molecular material using a Lorentz oscillator model, and can thus be calculated from directly measurable macroscopic quantities. The maximum splitting that can be reached for a given material turns out to be the well-known value obtained for bulk polaritons,^66^ and is independent of cavity geometry.^64,65,67,68^

While the available Rabi splittings are similar, the two types of setups have complementary strengths and weaknesses and, thus, serve quite different uses. As commented above, optical microcavities are characterized by large mode volumes and thus require macroscopic numbers of molecules to achieve strong coupling, with typical values ranging from 10^6^ to 10^10^ molecules per cavity mode^33,69,70^ at optical frequencies and even more at IR frequencies under vibrational strong coupling. The polaritonic modes are then delocalized over many molecules, giving rise to collective effects and effective long-range interactions between spatially separated molecules.^2,3,16−19,21−31^ While there is a wide range of designs that have been developed for optical light confinement,^71−74^ experiments in polaritonic chemistry have almost exclusively used Fabry–Pérot cavities consisting of two planar mirrors. The mirrors are typically either made of metal or from distributed Bragg reflectors (DBRs, alternating layers of dielectric materials with different refractive indices). Metal mirrors are easier to fabricate but, at optical frequencies, lead to quite lossy cavity modes with low quality factors (*Q* ≈ 10), where *Q* = ω_c_/κ is the ratio between the cavity mode frequency ω_c_ and its decay rate κ, and corresponding lifetimes τ = 1/κ on the order of 10 fs. In contrast, DBR mirrors can be fabricated with relatively high reflectivity and low losses, giving quality factors on the order of *Q* = 1000 and cavity mode lifetimes on the picosecond scale.

Subwavelength nanocavities also feature a very large flexibility in the design, with the field confinement being tunable through the size and shape of the plasmonic platform.^75^ The large confinement typically leads to a strongly inhomogeneous EM field profile,^76−78^ in particular, when atomic extrusions form so-called picocavities.^53,79^ This makes accurate placement of the emitters crucial, which can, for instance, be achieved through the use of DNA origami.^51,80,81^ Due to the intrinsic losses present in metals,^82^ plasmonic nanocavity modes are limited to short lifetimes (typically below 10 fs),^83^ such that most dynamics become dominated by ultrafast radiative and nonradiative decay. While this poses a challenge for polaritonic chemistry approaches that rely on dynamics in the excited state, these fast losses can also be exploited to open up additional relaxation channels that can be beneficial for the desired application, such as photoprotection,^11−13,16^ suppression of undesired side reactions,^18^ opening of new reaction channels,^14^ sensing applications,^84^ and imaging techniques for ultrafast processes.^85^

## Theoretical Approaches and Challenges

As the above discussion shows, theoretical approaches aimed at describing the rich phenomenology of molecules strongly coupled to confined EM modes encounter an inherently multiscale problem, with distinct challenges depending on which type of situation is to be treated: large ensembles of molecules with collective effects and long-range phenomena (in optical microcavities) or few molecules interacting with a complex, highly lossy and inhomogeneous electromagnetic environment (plasmonic nanocavities). In this section, we discuss the principal aspects and approaches that have been developed over the past few years to treat such systems.

The “correct” theory for describing molecules is nonrelativistic quantum electrodynamics (QED),^86,87^ which describes the interaction between charged point particles (electrons and nuclei) and EM fields. In general, the coupled Hamiltonian (in Coulomb gauge) can be written as
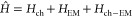
2where *H*_ch_ describes the kinetic energies and Coulomb interactions of the charged particles, *H*_EM_ describes the radiative (transversal) EM field modes (which are harmonic oscillators), and *H*_ch–EM_ describes the interactions between charges and EM modes. In free space (and in the absence of external driving fields), the interaction between light and matter is weak and its main effect is the radiative decay of excited states due to the spontaneous emission of photons (excitations of the free-space EM field). The standard approach of quantum chemistry is thus to only treat *H*_ch_ explicitly to obtain the approximate molecular energy structure (exact solutions are only possible for the very smallest molecules), and to either ignore spontaneous emission completely (when only short-time dynamics are of interest) or to treat it perturbatively. Typical spontaneous emission lifetimes for good molecular emitters (i.e., molecules with large transition dipole moments, μ ∼ 10 D) at optical frequencies are on the order of a few nanoseconds, with some *J*-aggregates (where a collective excitation is distributed over *N* monomers) reaching down to tens of picoseconds at cryogenic temperatures.^88,89^ This is slow compared to vibrational relaxation and thermalization, which typically happen on subpicosecond to few-picosecond scales.^90^ In cavities, the role of the EM field becomes more relevant, and the assumption that *H*_ch_ can be treated separately breaks down when the light–matter interaction becomes strong enough. It then becomes necessary to also treat *H*_EM_ and *H*_ch–EM_ explicitly to obtain the correct energies and states of the coupled system. Therein lies the rub of polaritonic chemistry.

Before turning to more practical considerations, we point out that, in the above statement about the importance of EM modes in cavities, we have silently changed the concepts we are using by pretending that a “cavity” is an abstract way of changing the EM mode Hamiltonian. In line with this useful lie, cavity modes are often described as arising from applying boundary conditions to the EM field modes. However, in reality, any cavity is a material system, that is, a collection of charged particles (such as mirrors or plasmonic nanoantennas) that are arranged so as to influence the EM field modes and to achieve the desired properties. It is thus more correct to perform a repartitioning *Ĥ*, with the parts of *H*_ch_ and *H*_ch–EM_ describing the cavity material and its interaction with the EM field being grouped with *H*_EM_ and forming a new “cavity” Hamiltonian *H*_cav_, such that
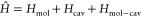
3where *H*_mol_ is now only the molecule (or any other material system) that will be treated in detail, while *H*_cav_ describes the combined excitations of the coupled cavity material and free-space EM modes. Under the assumption that the cavity material can be treated through linear response, diagonalizing *H*_cav_ is equivalent to solving the macroscopic Maxwell equations (see ref (91) for an overview). It is in this sense that *H*_cav_ is often said to describe the EM field, and its excitations are called “photons”. In particular, its eigenmodes keep being harmonic oscillators. However, ignoring the simple fact that *H*_cav_ also includes a material response can have serious consequences and lead to misleading conclusions. For example, plasmonic nanocavity modes mostly correspond to material excitations (collective oscillations of the electrons in the metal), and their interaction with the molecules are mostly mediated by (longitudinal) Coulomb interactions, not by (transversal) free-space EM modes. The Coulomb interaction is not affected by the Power–Zienau–Woolley transformation and, in particular, gives an *E⃗*·*d⃗* interaction, even in minimal coupling, without any dipole-self-energy term.^91,92^ The dipole-self-energy term should thus not be included when treating a physical situation corresponding to a strongly subwavelength (e.g., plasmonic) nanocavity, which is the only available way to approach single-molecule strong coupling. Results in the literature with single-molecule strong coupling, where the dipole self-energy term is included, should therefore be approached with care.

As mentioned above, when assuming a linear response for the cavity material, *H*_cav_ can be diagonalized as a collection of harmonic oscillators, just like the free-space EM field. Formally, there is always a continuum of solutions existing at any (positive) energy. In practice, this can often be reduced to an effective description where only a single or a few “cavity modes” have to be treated explicitly, although the coupling to the residual continuum means that these cavity modes are generally resonances with finite (and possibly very short) lifetimes.^93−95^

After these general considerations, which are normally skipped over in the literature (which has to be done with care, as discussed above), we have thus finally arrived at the Hamiltonian that is often the starting point in the literature on polaritonic chemistry. We now discuss available approaches for solving the Hamiltonian, eq 3, which describes three types of degrees of freedom: electronic (**r**), nuclear (**R**), and photonic (**q**). Depending on the level of description with which each of its terms is treated, we can roughly categorize the numerous methods available in literature by their level of realism, as sketched in Figure 1. We note that for consistency, we write the cavity modes using the “position space” degrees of freedom **q**. The Hamiltonian of a cavity mode with frequency ω_c_ is , which can equally be expressed in terms of the ladder operators, , giving . This form is typically used in quantum optics, as it allows a natural interpretation of the operators *a* and *a*^†^ as annihilating and creating a photon, respectively.

**Figure 2:**
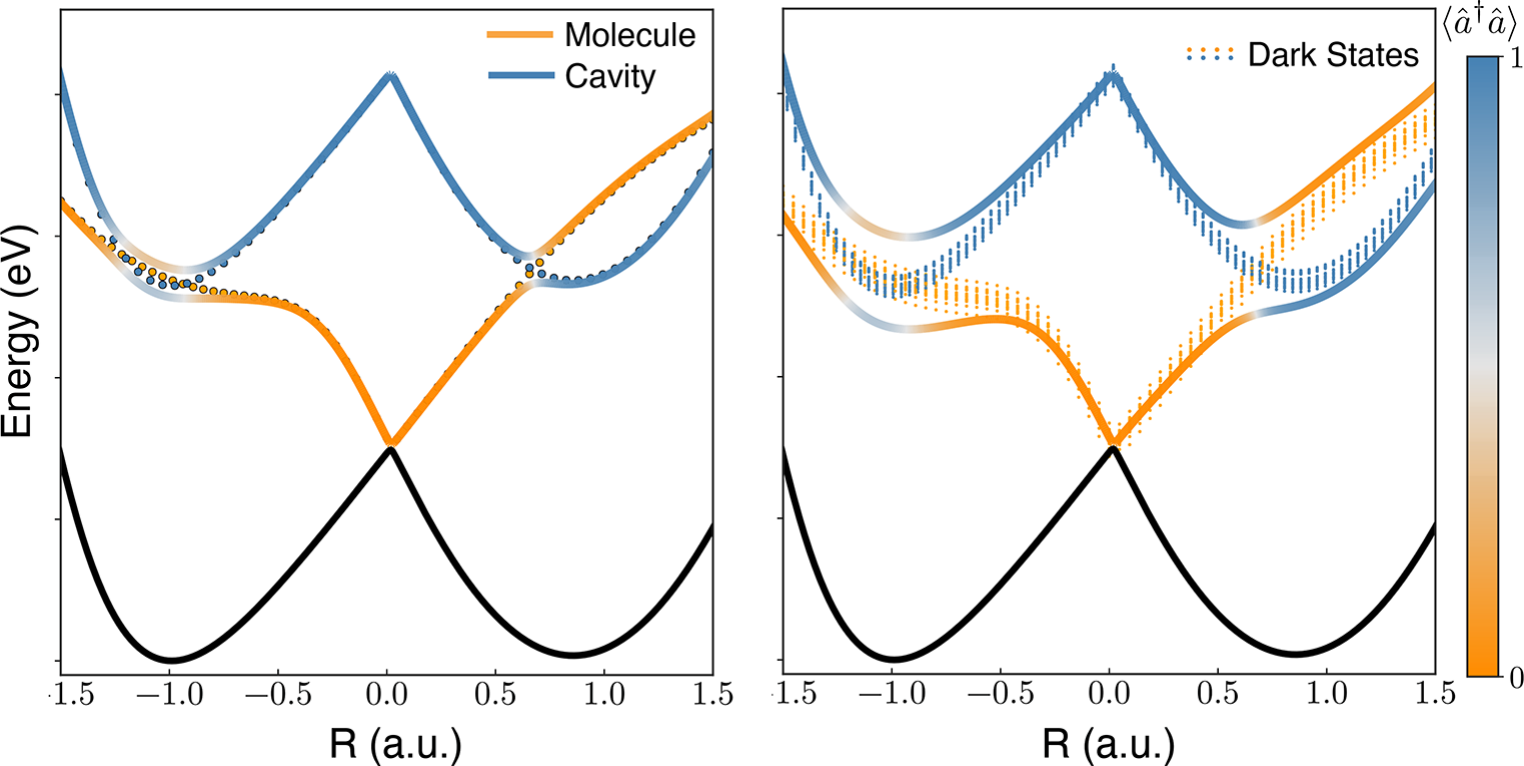
Polaritonic potential energy surfaces. (a) Case of a single molecule strongly coupled with light, where coupling between the cavity (blue dotted) and the molecule (orange dotted) states couple to originate polaritons. (b) Case of a molecular ensemble (*N* = 50), where a manifold of dark states emerges.

When treating a system described by the Hamiltonian (eq 3), it can be helpful to factorize the time-dependent wave function Ψ(**r**, **R**, **q**, *t*) using a Born–Huang expansion, where slow and fast degrees of freedom are separated. In electronic strong coupling, which we focus on here, the cavity mode frequencies are (close to) resonant with electronic transitions, and the dynamics of electrons and cavity modes are thus comparably fast, making it natural to group them together^8,96,97^
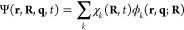
4Here, the states ϕ_*k*_ (**r**, **q**; **R**) are the eigenstates of the Hamiltonian without the nuclear kinetic energy. They are mixed photonic and electronic (polaritonic) states that parametrically depend on the nuclear coordinates, with the associated energies being polaritonic potential energy surfaces (PoPES).^4^ Potential energy surfaces as a tool are extensively used to simulate and predict the properties and outcomes of photochemical reactions. As such, the adaptation of this tool to polaritonic chemistry can describe how the energy landscape, and consequently the reactivity, is modified when molecules are brought into strong coupling. By coloring the PoPES according to the projection of the polaritonic eigenstates on the electronic and photonic subspace, they can also directly be used to obtain information about whether the excitation on a given surface is more photon- or more exciton-like, that is, whether the energy is stored in the cavity or in the molecule, as sketched in Figure 2a. Typically, a single PoPES polaritonic state will gradually change its character as a function of nuclear coordinate. This can lead to periodic transfer of energy between the molecule and cavity due to nuclear motion in a process that is completely distinct from conventional vacuum Rabi oscillations and could allow, for example, following the nuclear wave packet motion in time.^85^

We note that it is also possible to use exact factorization methods for analyzing the cavity-induced molecular dynamics^98^ or to group the photonic and nuclear coordinates together, such that electronic states parametrically depend on the photonic and nuclear coordinates **q**, **R**, leading to the so-called cavity Born–Oppenheimer (CBO) approximation.^99^ This approach is especially powerful in the regime of vibrational strong coupling (VSC), where nuclear motion and photonic dynamics are comparably fast, and the dynamics usually takes places on the lowest (ground) electronic state.^92,100^ The problem to be solved is then that of dynamics on a single high-dimensional PES, which is conceptually the same as for “normal” out-of-cavity ground state reactions. For such problems, semiclassical approximations are well-understood and usually applicable. In contrast, the CBO is not ideal for describing electronic strong coupling, in which hybridization occurs between electronic and photonic degrees of freedom and separating them conceptually does not provide a natural framework. To be precise, polariton formation then requires hybridization between nuclear + photonic sublevels on different electronic surfaces. Whether this occurs is not obvious from visual inspection of the surfaces. For numerical implementations, this process is also not well-described by semiclassical techniques and thus requires a full quantum description of nuclear + photonic motion, negating most of the advantages of using a Born–Oppenheimer-like approach in the first place.

In order to obtain the PoPES and the nonadiabatic couplings between them, it is thus necessary to solve the coupled electron–photon Hamiltonian. There are two main strategies that have been followed to achieve this, both of which are formally exact and ab initio, but have different strengths and weaknesses. To aid the reader in navigating the theoretical approaches, we report a visual guide in Table 1. The first is conceptually comparable to a configuration interaction (CI) approach where the Hamiltonian is first diagonalized without including the light–matter interaction, and the eigenbasis of the uncoupled Hamiltonian is then used to express and diagonalize the full Hamiltonian. This approach has several clear advantages. On the one hand, it is quite straightforward to implement, as it allows the use of any of the methods in the toolbox of standard quantum chemistry (QC) to solve the molecular problem. If the light–matter coupling is treated in the commonly used dipole (or long-wavelength) approximation, only the electronic energies and (permanent and transition) dipole moments have to be calculated. We note that permanent dipole moments are often disregarded in the literature, which implicitly corresponds to assuming that the permanent dipole moment is approximately independent of electronic state and nuclear position, which is not necessarily a good approximation. Higher-order light–matter couplings, such as quadrupolar interactions^77^ can also be included if the quadrupole moments are calculated. Second, it allows for an easy interpretation of the resulting polaritonic states, as they are expressed as superpositions of the physical eigenstates of the uncoupled system with well-defined properties. Finally, the convergence of the approach can be tested by including successively more electronic states and is usually quite rapid, especially when the per-molecule coupling strength is not too large. In particular, it is often sufficient to only include two electronic states (the ground and first excited state). In the literature, a wide range of quantum chemistry (QC) methods have been employed to provide the input for this CI-like treatment of polaritonic chemistry, such as TDDFT,^96^ semiempirical methods,^97^ MRCI,^101^ and CASSCF.^102^

**Table 1:**
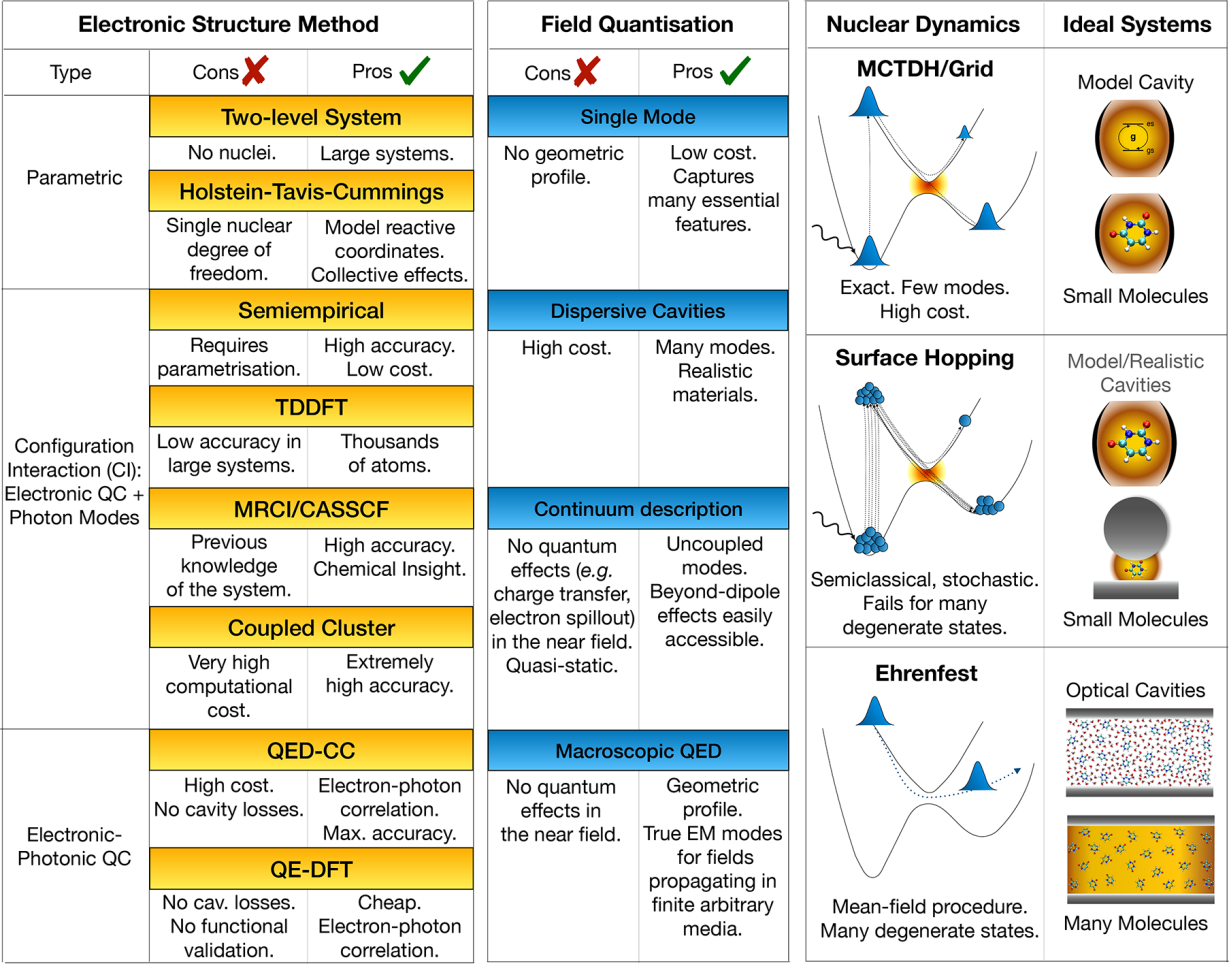
Summary of the Theoretical Approaches for Polaritonic Photochemistry^a^

a The level of description of polaritonic states depends on the description of the uncoupled subcomponents (electronic and photonic). The QED-CC and QE-DFT, instead, compute directly the polaritonic states without resorting to the uncoupled states. The polaritonic calculation can then be paired with the nuclear dynamics techniques reported on the right-hand side of the table.

The second strategy to treat light–matter coupling within the electron–photon Hamiltonian relies on extending QC methods to directly include cavity modes in their solution. The advantages of these approaches are that they are expected to more easily capture changes in state wave functions that would require large expansions in the polaritonic CI approach discussed above. This becomes especially relevant when coupling strengths are large. Two notable developments in this direction are QE-DFT^103,104^ and QED-CC.^105,106^ The former is computationally cheap, but inherits the intrinsic problems of density functional theory approaches, since all known exchange and correlation functionals correspond to severe approximations.^107,108^ The latter offers a robust but computationally expensive alternative. As mentioned above, the strength of these approaches lies in the description of electronic–photonic states that are not just superpositions of closely lying uncoupled states, which happens for coupling strengths that are large enough. In the CI approach, convergence then requires the calculation of an enormous number of excited states. It is then at some point computationally cheaper and more straightforward to calculate the “new” electronic–photonic states directly instead of using the uncoupled states as the expansion basis. However, it should here be noted that single-molecule changes usually depend on the single-molecule coupling strength and are not collectively enhanced in many-molecule setups.^8,109,110^ This effect is thus not expected to be present in such systems, and few-state expansions should work well. In contrast, for the largest single-molecule coupling strengths available (in plasmonic nanocavities with gaps on the order of 1 nm^51^), treating the cavity mode as a lossless photonic mode and neglecting the atomistic structure of the plasmonic nanocavity are both severe approximations.^111,112^

Once the method to obtain the polaritonic (electronic–photonic) structure of a given problem has been chosen, some way to treat the nuclear motion has to be included. The cheapest method is to not do any nuclear dynamics, that is, to simply analyze the obtained PoPES. This can already provide significant insight about the possible changes in the system response due to strong coupling but, of course, precludes any quantitative insight. Going beyond this, semiclassical methods based on surface hopping are powerful tools,^9,10,12,96^ as they can qualitatively describe a large number of nuclear degrees of freedom when a relatively small number of excited states is involved. As such, they are best exploited to describe one to a small number of molecules, as the algorithm fails at grasping collective effects even in the more refined implementations.^113−116^ The failure is due to the inaccurate evaluation of transition probabilities in the presence of many quasi-degenerate states,^117^ which is exactly the case typically encountered when many molecules couple to a single cavity mode.^4,118^ An additional problem for the current implementations of semiclassical algorithms that may be potentially hindering to polaritonic chemistry is the incapacity of describing tunneling through potential energy surfaces. A palliative solution to this problem comes from partially including the nuclear quantum effects in the semiclassical simulations, for example, with the ring polymer technique.^119,120^ One big advantage that semiclassical techniques offer is that it becomes easier to include more of the environmental complexity, such as atomistic descriptions of the solvent^10^ and chemical environment,^96^ achieved by including the electrostatic interactions between classical MM charges and the QM charge density (electrostatic embedding). Furthermore, trajectory-based approaches^121^ allow the straightforward inclusion of cavity losses via quantum jump algorithms^122,123^ in the framework of the stochastic Schrödinger equation (SSE)^124−126^ and non-Hermitian formulations.^12,127^

As a counterpart to semiclassical techniques for the treatment of nuclear motion, quantum wavepacket dynamics can provide highly accurate results for a restricted number of degrees of freedom with the drawback of a much larger computational cost. For low-dimensional model problems, direct grid-based methods are relatively straightforward to implement and provide accurate solutions.^7,8,16,128^ For high-dimensional nuclear wave functions, the method of choice is the multiconfigurational time-dependent Hartree (MCTDH) algorithm,^129,130^ possibly in its multilayer implementation.^131^ When potential surfaces can be approximated as harmonic oscillators, tensor network approaches are another powerful way to perform full quantum dynamics.^132,133^ As a hallmark feature, methods relying on wavepacket propagations guarantee the correct dynamics of the nuclear wavepacket at both electronic and polaritonic avoided crossings, conical intersections, and seams between the PoPESs, including a correct decay of nuclear coherence without needing to resort to artificial corrections, as in the semiclassical methods. Second, its propagation allows to exactly include decay channels in the dynamics, either through effective non-Hermitian Hamiltonians^11,12,85^ that are exact when the dynamics after decay are not of interest or by direct solution of a Lindblad-style master equation.^13,14^ This feature is particularly advantageous when the polaritonic relaxation involves multiple polaritonic states and the decay mechanism is an interplay between radiative and nonradiative transitions. These characteristics make wavepacket dynamics an excellent investigation tool to explore the effect of cavity losses or the role of strong coupling on conical intersections.^134−136^

The propagation schemes for nuclei have proven instrumental in surveying new effects and predicting new applications when few molecules are involved. Among them, we count the suppression/enhancement of photoisomerization reactions, photoprotection/photostability of organic chromophores,^11−13,16,137^ photodissociation,^7,14,128,138^ and reverse intersystem crossing (RISC).^33,37^

A common approximation in the methods discussed above is to rely on a single cavity mode. An extension to the case of multimodal cavities has been implemented only recently.^29^ Furthermore, only few approaches have tried to combine a quantum chemical description of the molecule with a realistic nanophotonic setup. These approaches rely on the quantization of the electromagnetic environment via different approaches.^11,139^ It is an open question and important challenge to understand whether such approaches are valid in the limit of atomistic resolution that is approached in recent experiments in nanoplasmonic gap cavities^140,141^ even though they rely on continuum descriptions of the cavity (plasmonic) medium. There are encouraging indications that this is possible.^52^ As such, these methods will be potentially able to guide the investigation of polaritonic chemistry in setups confining the electromagnetic field at subnanometric volumes, such as picocavities.^53,79^

Despite the accurate level of description reached for strong coupling in few-molecule problems, the modeling of polaritonic reactions meets an intrinsic problem when trying to describe large ensembles. Most of the polaritonic chemistry experiments are performed in microcavities, where up to *N* = 10^10^ emitters are involved. In principle, the PoPESs in such a setup are *N* × *N*_*m*_-dimensional, where *N*_*m*_ is the number of nuclear degrees of freedom required to describe a single molecule (possibly including the chemical environment). If the molecules were decoupled, the strategy would be to treat a restricted number of molecules (one to few) via quantum chemistry methods, including the chemical environment molecules (solvent or protein scaffolds) atomistically (QM/MM techniques) or as a continuum medium (PCM techniques).^142^ Instead, the strongly delocalized electromagnetic field in the cavity opens up long-range interaction channels in a disordered ensemble of molecules.^143^ This makes it highly challenging to infer photochemical properties of an ensemble of *N* molecules from the detailed study of a very restricted subset of it. To take into account the large number of emitters, one approach is to use strongly simplified molecular models, such as the Holstein model where each molecule is described by two displaced harmonic oscillators describing nuclear motion in the electronic ground and excited states. This allows including a large (few thousands) number of molecules, coupled to the cavity with a Tavis–Cummings-like model.^42^ Despite its success in predicting long-range energy transfer^25,26^ and remote catalysis,^25^ the exciton-based approaches present several drawbacks. The most evident is that the nonatomistic description does not allow to grasp structural rearrangements of molecules upon, for example, charge transfers and the associated chemical environment rearrangement. This can be included by approaching the problem of collective effects using multiscale techniques.^27,96,144,145^ The approach initially developed by Luk et al.^96^ already implements a QM/MM description of molecules in cavities and has been extended to a multimode cavity characterized by a 1D dispersion.^29^ Its current implementation already supports a large number of both wave function and density functional methods, interfaced with both surface hopping and Ehrenfest dynamics.^96^ In the presence of many molecules and thus a large manifold of closely spaced PoPES, Ehrenfest dynamics provide more robust results compared to surface-hopping approaches.^27^

**Figure 3:**
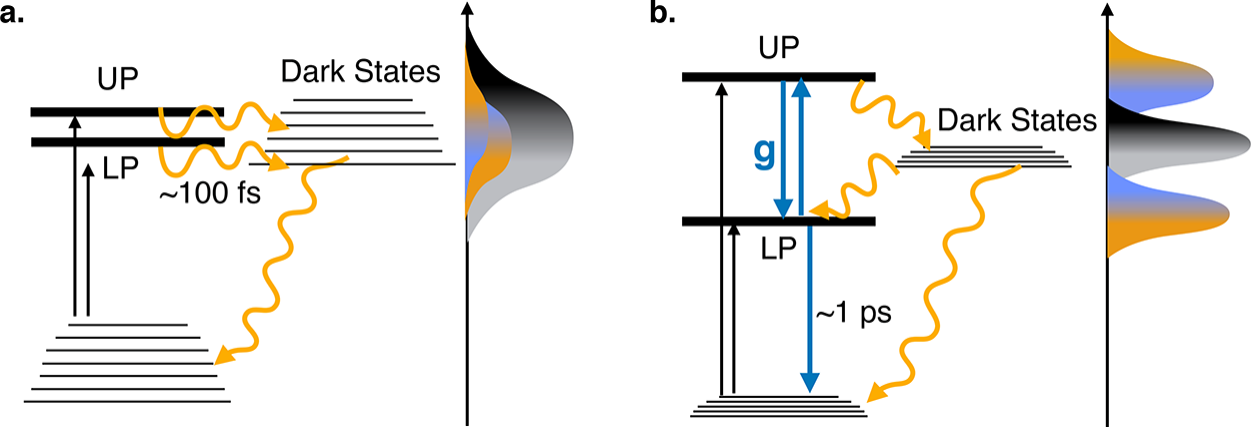
Dominant processes in polaritonic systems. (a) Ultrafast decoherence of the cavity excitation. The overlap between polaritonic bright and dark states funnels the wavepacket toward the dark manifold, where the wavepacket undergoes decoherence via nonradiative processes of the individual molecules. (b) Dominant processes occur from the lower polaritonic state, as the dark manifold are decoupled from the polaritonic states. This scheme implies a long-lived delocalized excitation, which can potentially result in a cavity-modified chemistry.

A prominent signature of the necessity to describe large ensembles is the emergence of a dark state manifold when ensembles of molecules are coupled to a cavity (Figure 2b). Within the first excited subspace, there are *N* + 1 states, each of them corresponding to a single excitation (either in one of the *N* molecules or in the cavity mode) of the global system from its ground state. The so-called bright state is obtained when the molecular excitation is delocalized over all the resonant molecules. This state couples ideally to the cavity mode (with effective coupling enhanced by √*N* over the single-molecule one). The molecular bright state and the cavity mode couple to form the typical upper and lower polariton modes. In a simple conceptual picture, all the other orthogonal superpositions obtained by distributing a single excitation over the molecules constitute the *N* – 1 dark states manifold. We note that this simple picture is only true in the case of perfectly degenerate two-level emitters,^146^ but it provides a convenient framework to think about the states in the system. In particular, when the molecules are not identical (or the nuclear configurations are distinct, even for nominally identical molecules), the dark states are not fully dark and provide residual light absorption and emission. While it is conceptually common to think about the dark states as states in which the excitation is localized on individual molecules, it has been shown that the dark state manifold inherits some of the delocalized polaritonic properties.^147,148^ Still, the energy distribution of dark states closely matches the absorption spectrum of the bare molecules.^27,149^ Furthermore, the potential energy landscape of each of these states (Figure 2b) looks quite similar to the collective ground state of the isolated molecular ensemble. The role of dark states in polaritonic processes is then strongly dependent on the specifics of the system: when the dark states manifold embeds (strongly overlaps with) polaritonic states, which in particular happens for broadband absorbers,^40,102^ the polaritons dephase into cavity-free superpositions of states in the dark manifold. This ultrafast loss of coherence to the dark states can become the dominant decay process for polaritons^27^ (see Figure 3a), resulting in reactivity essentially equal to that of isolated molecules.^102^ Put in another way, if we want to ensure that photochemical reactions can efficiently take place on the polaritonic potential energy surfaces, the Rabi splitting should be larger than the molecular absorption band. We note that the lifetime of the polaritons is not limited by the molecular absorption bandwidth since the latter is dominated by the spread of molecular excitation energies, not by the intrinsic lifetime of molecular excitations.^146^ This implies that there is no reason to “match” the cavity bandwidth to the molecular absorption band, and indeed, when the polaritons do not overlap with the dark states, the dominant decay process becomes radiative decay from the lower polariton (Figure 3b). This occurs at roughly half the bare-cavity decay rate (which can translate to lifetimes from the few-femtosecond to picosecond range), and can give line widths much smaller than the bare molecular one.^61^ Such decay times are comparable to those of several photochemical reactions,^150^ confirming the possibility to influence photochemistry with polaritons. A further important parameter is the Stokes shift or reorganization energy of the molecules, which describes the energy shift between the relaxed ground- and excited-state nuclear configurations. When this is large enough for the excited-state energy minimum to lie below the lower polariton energy, it opens a new nonradiative relaxation channel.^151^ The same effect is also present when other electronic states lie below the polariton energy.

## Conclusions and Outlook

Over the past years, polaritonic chemistry has developed into a vibrant field that is drawing increasing attention both from the experimental and theoretical communities. It holds the promise of providing an approach to control (photo)chemical reactions that is completely distinct from traditional ones and, in particular, does not rely on the external input of energy apart from absorption of single photons. The theoretical description of these processes faces many challenges due to the inherently multiscale nature of the problem, with unique challenges arising in each of the two distinct types of common experimental setups. In wavelength-scale optical cavities, the macroscopic number of participating molecules a priori prevents a full representation of experimental reality in the theoretical approaches, as the sheer number of degrees of freedom of the problem poses serious challenges even to semiclassical approaches. Furthermore, there is usually a continuum of EM modes that has to be taken into account for obtaining a complete picture. While experimentally much simpler to construct than nanoplasmonic resonators requiring (sub)nanometric precision, the theoretical treatment of cavity-modified molecular reactions in wavelength-scale optical cavities thus faces a plethora of challenges and will require the judicious use of appropriate approximations.

In subwavelength cavities with single- or few-molecule strong coupling, accurate descriptions are challenged by the large loss rates, the complex nature of the EM field modes, and the importance of atomistic details in the material structures providing the cavity modes. One way forward here will be given by methods able to quantize the plasmonic electromagnetic field in arbitrary material structures^94,95,139^ and their interface with quantum chemistry methods and nonadiabatic dynamics techniques to account for the molecular reactivity. Going further, the inclusion of quantum effects such as tunneling at the nanoparticle–molecule interface calls for a multiscale layered technique, where the interface has to be described at a quantum-mechanical atomistic level, while still taking into account the global EM modes and plasmonic excitations.

In addition to methodological challenges, there are also significant experimental and conceptual obstacles to overcome on the path toward actual devices based on the concepts of polaritonic chemistry. As an example, strategies to either exploit or minimize losses are required, particularly in subwavelength plasmonic cavities. There, the capability to reach longer lifetimes would open up new intriguing phenomena taking place at the picosecond time scale. One promising approach here could be provided by hybrid metallodielectric cavities (see ref (95) and references therein), in which plasmonic excitations are hybridized with long-lived optical cavity modes, allowing to control the trade-off between strong field confinement and material losses in metals. Another possibility that has not yet been explored in this context are purely dielectric nanophotonic cavities designed to achieve subwavelength field confinement while still largely avoiding losses.^74^ In parallel, it remains to be seen whether the use of atomic-scale extrusions (leading to picocavities) can enable control over chemical reactions on the single-molecule level, possibly even with subnanometer precision.

As in many previous cases of theoretical investigation, this search for theoretical and numerical frameworks able to accurately describe the physical and chemical process emerging in polaritonic chemistry at very different scales will not only lead to a better understanding of the fundamental mechanisms involved in the current experimental setups and guide the exploration of new reliable platforms, but will also open new avenues for research in polaritonic chemistry and related areas that we cannot foresee at this stage.
